# The effect of pre-existing mental health comorbidities on the stage at diagnosis and timeliness of care of solid tumor malignances in a Veterans Affairs (VA) medical center

**DOI:** 10.1002/cam4.483

**Published:** 2015-06-09

**Authors:** Roxanne J Wadia, Xiaopan Yao, Yanhong Deng, Jia Li, Steven Maron, Donna Connery, Handan Gunduz-Bruce, Michal G Rose

**Affiliations:** 1 Yale School of Medicine, Yale UniversityNew Haven, Connecticut; 2 Veterans Affairs Healthcare SystemWest Haven, Connecticut; 3 Yale School of Public Health, Yale UniversityNew Haven, Connecticut; 4 University of ChicagoChicago, Illinois

**Keywords:** Colorectal neoplasms, head and neck neoplasms, mental health, neoplasm staging, quality of health care, time factors, urologic neoplasms, veterans health

## Abstract

There are limited data on the impact of mental health comorbidities (MHC) on stage at diagnosis and timeliness of cancer care. Axis I MHC affect approximately 30% of Veterans receiving care within the Veterans Affairs (VA) system. The purpose of this study was to compare stage at diagnosis and timeliness of care of solid tumor malignancies among Veterans with and without MHC. We performed a retrospective analysis of 408 charts of Veterans with colorectal, urothelial, and head/neck cancer diagnosed and treated at VA Connecticut Health Care System (VACHS) between 2008 and 2011. We collected demographic data, stage at diagnosis, medical and mental health co-morbidities, treatments received, key time intervals, and number of appointments missed. The study was powered to assess for stage migration of 15–20% from Stage I/II to Stage III/IV. There was no significant change in stage distribution for patients with and without MHC in the entire study group (*p* = 0.9442) and in each individual tumor type. There were no significant differences in the time intervals from onset of symptoms to initiation of treatment between patients with and without MHC (*p* = 0.1135, 0.2042 and 0.2352, respectively). We conclude that at VACHS, stage at diagnosis for patients with colorectal, urothelial and head and neck cancers did not differ significantly between patients with and without MHC. Patients with MHC did not experience significant delays in care. Our study indicates that in a medical system in which mental health is integrated into routine care, patients with Axis I MHC do not experience delays in cancer care.

## Introduction

Axis I mental health disorders encompass major psychiatric illness, including mood disorders (e.g., depression, bipolar disorder), anxiety disorders (e.g., post-traumatic stress disorder (PTSD)), psychotic disorders (e.g., schizophrenia, schizoaffective disorder), and addiction disorders such as drug and alcohol abuse. They affect approximately 43 million adults in the United States which is 18.3% of the population 1. The estimated prevalence of mental health comorbidities (MHC) among Veterans using the Veterans Affairs (VA) system is between 25% and 40% 2–4. The higher prevalence of MHC among US veterans in the VHA is thought to be due to increased exposure to risk factors such as trauma and combat, and the fact that veterans with MHC often use the VHA preferentially over the private sector 4,5. Among conditions treated yearly in the VA system, 11% are mental health illnesses (3.8% PTSD, 6.2% other mental/emotional problems and 1.2% drug and alcohol problems), and 7% are malignancy 6.

There have been several large population-based studies looking at cancer incidence and mortality among patients with MHC 7–13. Data from Australia has demonstrated an equal or decreased incidence of cancer in patients with MHC 12, whereas data from the US 7 and Denmark 8 have shown an increased incidence of malignancy in this population. In another US study, the mortality rates and years of potential life lost for public mental health clients were examined and, while they were found to die of the same causes as the general population, mental health patients had a higher relative risk of death than the general population 14. Studies done in Australia, Japan, France, the United States, and Denmark have all suggested that patients with MHC have higher cancer fatality rates. The studies based in France 9 and Japan 10 were focused on patients with schizophrenia, while the studies in Australia, Denmark and the United States looked at all patients with MHC. An Australian population-based study has shown that patients with MHC present later and with more advanced disease 15. This has also been suggested in studies of individual malignancies which have shown that MHC were related to delayed presentation and/or diagnosis in patients with esophageal 16, breast 17,18, lung 19, head and neck 20, and skin squamous cell cancers 21,22.

Increased mortality may also be caused by differences in the treatment delivered. The Australian study showed that patients with psychiatric illness were less likely to undergo surgery, and they receive less chemotherapy or radiotherapy sessions 15. Women with breast cancer and depression were less likely than their counterparts without depression to accept adjuvant treatment 21. Physician adherence with breast cancer chemotherapy regimens, however, was not found to be affected by the presence of a psychiatric comorbidity 23. Veterans with esophageal cancer and psychiatric comorbidities were less likely to undergo surgery, although it was unclear if this was due to patient noncompliance or provider bias in offering esophagectomy 16. In severe psychiatric disease where capacity to make medical decisions is affected, treatment may not be initiated in a timely manner due to a lack of understanding on the part of the patient and the inability to secure a surrogate decision maker 24. There is limited data on the impact of MHC on stage at diagnosis and timeliness of care in patients with cancer.

The purpose of this study is to examine the initial stage at diagnosis for patients with and without MHC, determine whether there is a difference in the timeliness of care between patients with and without MHC, and establish if MHC is associated with patient-mediated delays in care in Veterans with newly diagnosed solid tumor malignancies at the Veterans Affairs Connecticut Health Care System (VACHS).

## Methods

**Figure 1:**
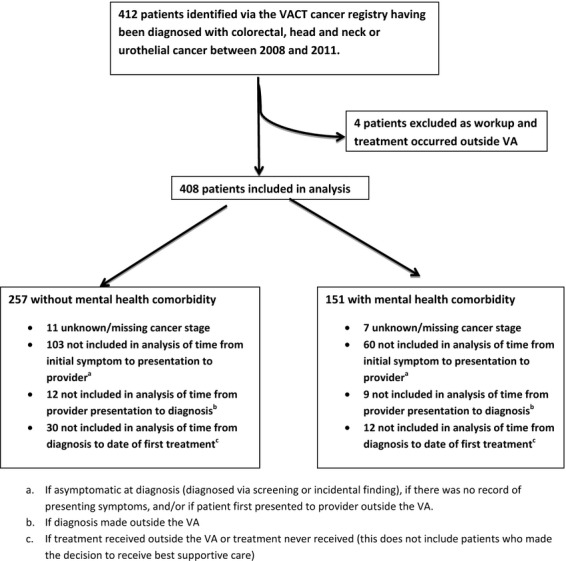
CONSORT diagram of chart inclusion, analysis.

This was a population-based retrospective cohort study using the electronic medical record and VA CT cancer registry data. The study was approved by the VACHS Institutional Review Board. The study population consisted of 412 veterans diagnosed with colorectal, head, and neck, or urothelial carcinomas between the years 2008–2011. These malignancies were chosen based on their prevalence at our institution. Prostate cancer was intentionally not examined as recommendations regarding prostate cancer screening and treatment had changed during the study period. Lung cancer data had been studied previously 19. Four patients identified by the cancer registry were excluded from our analysis as they had not received any of their care at the VA (Fig.1).

Patients were identified via the cancer registry. For each patient, demographic data, tumor characteristics, primary treatment, preexisting comorbid conditions, and time intervals were collected from the cancer registry, from ICD-9 codes and from review of progress notes. We captured the comorbid conditions that are known to impact timeliness of care 25. These included coronary artery disease, chronic obstructive pulmonary disease, diabetes, cerebrovascular accident, deep vein thrombosis or pulmonary embolism on active anticoagulation, chronic kidney disease, AIDS, cirrhosis, peripheral vascular disease, heart failure, atrial fibrillation, and other malignancies diagnosed within the 5 years previous to the current cancer diagnosis (excluding squamous or basal cell cancers of the skin), and connective tissue disease. The American Joint Commission on Cancer (AJCC) 7th edition, staging was used for all patients 26. DSM IV was used to define Axis I MHC 27. Patients were identified as having a preexisting Axis I mental health comorbidity if they had received this diagnosis via ICD-9 code and/or documentation in a provider note at least 1 year prior to the diagnosis of malignancy and had been seen by a mental health provider and/or received psychoactive medication. Data on alcohol and illicit drug abuse and dependence were collected separately.

The date of diagnosis was defined as the date that the histologic diagnosis of malignancy was made by a pathologist. The date of first symptom was the patient's self-reported date in which he or she first noted cancer-related symptoms as documented by review of the electronic medical record. If the patient was asymptomatic and the malignancy was found via screening or incidentally, this date was not collected and these patients were not included in the analysis of the time interval from the first symptom to its reporting to a provider. However, these patients were included in the other time interval measures. The date of the first provider encounter was considered the first documented date the patient reported the cancer-related symptom to a staff member in any clinical setting (including a phone call). The date of first treatment was defined as the date of surgery, or the first date of chemotherapy or radiation, depending on the first treatment received. If the first treatment was not administered at the VA, this date was not collected. In patients who were managed by supportive care only (whether by the provider or by patient choice), the date of first treatment was defined as the date in which this discussion was held with the patient. Three time intervals were calculated: time from date of first symptom to date of first presentation to provider, time from date of presentation to provider to date of diagnosis, and time from date of diagnosis to date of first treatment. A patient-related delay in care was defined as greater than a 14-day delay in a diagnostic procedure or in initiation of the first treatment caused by patient-related factors (e.g., patient declined an offered appointment, patient rescheduled an appointment, and/or patient missed an appointment). This information is available in the VA EMR appointment list (e.g., “no show,” “cancelled by patient,” “cancelled by clinic”). Chart abstraction was also performed to determine if the standard of care therapy, as defined by the National Comprehensive Cancer Network guidelines, had been offered to the patient, and if there were patient-related deviations from standard of care treatment. All charts were reviewed by one of two investigators (RJW, SM) and controversies were resolved by discussion and consultation with other investigators (MGR, JL).

With a sample size of 408, of which 151 had MHC, this study had 87% power to detect a 15% difference in the proportion of stage II/III cancer between patients with and without MHC, assuming that 25% of patients without MHC had stage I/II cancer and a two-sided 0.05 type I error. Patient characteristics were compared between patients with and without MHC by using Wilcoxon rank sum test for continuous variables and chi-square or Fisher's exact test for categorical variables. Analysis was performed by using SAS 9.3 (SAS Institute Inc., Cary, NC). A two-sided *P* value of 0.05 was used as the criteria for statistical significance.

## Results

A total of 408 patient charts were reviewed, of which 151 (36.6%) had Axis I MHC. Of the patients with MHC, 78% had a mood disorder (depression, bipolar disorder, anxiety or PTSD), 13% had dementia or cognitive impairment, and 8% had a psychotic disorder (Table1). Patients with or without MHC did not differ in regard to primary cancer site, number of medical comorbidities, stage at presentation, or percent of patients diagnosed with asymptomatic disease. Patients with MHC were more likely to be diagnosed at a younger age (median age of 64 vs. 70 years, *p* < 0.0024) and to use illicit drugs (*p* < 0.0001).

**Table 1:** Baseline characteristics of the study population

	Overall population (*N* = 412)	Without MHC (*N* = 257)	With MHC (*N* = 151)	*p*-value
Male gender (*N*, %)	407 (98.79)	255 (99.22)	148 (98.01)	0.3639
Race (*N*, %)				
Black	51 (12.38)	28 (11.02)	22 (14.86)	0.1754
White, other	354 (85.92)	226 (88.98)	126 (85.13)	
Median age at diagnosis (years)	67	70	64	0.0024
Primary cancer site (*N*, %)				
Urothelial	174 (42.23)	114 (44.36)	57 (37.75)	0.18
Colorectal	155 (37.62)	89 (34.63)	66 (43.71)	
Head and neck	83 (20.15)	54 (21.01)	28 (18.54)	
Stage at diagnosis-all cancers (*N*, %)				
0	118 (28.92)	76 (29.57)	42 (27.81)	0.9442
I–II	115 (28.19)	71 (27.63)	44 (29.14)	
III–IV	157 (38.48)	99 (38.52)	58 (38.41)	
Unknown	18 (4.41)	11 (4.28)	7 (4.64)	
Alcohol abuse/dependence (*N*, %)	77 (18.69)	41 (15.95)	36 (23.84)	0.0660
Illicit drug abuse/dependence (*N*, %)	21 (5.10)	4 (1.56)	17 (11.26)	<0.0001
Number of medical comorbidities (*N*, %)				
0–1	221 (53.64)	134 (53.60)	85 (56.67)	0.4098
2–4	171 (41.50)	107 (42.80)	63 (42.0)	
>4	96 (23.30)	9 (3.60)	2 (1.33)	
Cancer diagnosed at VHA (*N*, %)				
Y	346 (83.98)	216 (85.04)	130 (86.67)	0.7692
N	62 (15.05)	38 (14.96)	20 (13.33)	
Cancer symptomatic at diagnosis (*N*, %)				
No	123 (29.85)	76 (31.15)	47 (32.41)	0.8221
Yes	268 (65.05)	168 (68.85)	98 (67.59)	

MHC, mental health comorbidity; VHA, Veteran's Health Administration.

There was no significant change in stage distribution for patients with and without MHC in the entire study group and in each individual tumor type (Table1). Furthermore, the two groups did not differ regarding the three key time intervals: time from onset of symptoms to presentation to provider, time from presentation to provider to diagnosis, and time from diagnosis to initiation of treatment (Table2). Patient-related delays in care or deviations from standard of care occurred at the same rate in the patients with or without MHC (Table3). In a subgroup analysis looking at patients with alcohol and/or illicit drug abuse, we found no significant difference in any of the timeliness intervals when compared to patients without alcohol/illicit drug abuse.

**Table 2:** Timeliness intervals in patients with and without mental health comorbidities

	Without MHC; days (range)	With MHC; days (range)	*p*-value
Median time from symptoms to provider presentation (*N* = 245)	14 (0–1446)	9 (0–3287)	0.1135
Median time from provider presentation to diagnosis (*N* = 387)	22 (0–851)	26 (0–1490)	0.2042
Median time from diagnosis to treatment (*N* = 366)	44 (0–652)	43 (0–408)	0.2352

MHC, mental health comorbidites.

**Table 3:** Patient mediated deviations from standard of care in patients with and without mental health comorbidities

	Without MHC	With MHC	*p*-value
Patient mediated diagnostic delay (*N* = 368, %)			
No	187 (80.26)	108 (80)	0.99
Yes	46 (19.74)	27 (20)	
Patient mediated treatment delay (*N* = 380, %)			
No	205 (85.42)	117 (82.98)	0.7909
Yes	34 (14.58)	24 (17.02)	
Patient deviation from standard of care treatment (*N* = 389, %)			
No	212 (86.18)	116 (81.82)	0.1857
Yes	34 (13.82)	27 (18.18)	

MHC, mental health comorbidities.

Since patients with MHC in our cohort were significantly younger than patients without MHC, we examined the impact of age at diagnosis on the cancer presentation and the stage at diagnosis. Age at diagnosis did not differ between symptomatic and asymptomatic patients in the overall study population, or in the head and neck and urothelial cancer sub-groups. In the colorectal cancer group, asymptomatic patients were younger than symptomatic patients (66 vs. 69 years, *p* = 0.038). However, patients younger than 65 years of age were significantly more likely to present with later stage disease than patients aged 65 or older (30.59% vs. 18.41%, *p* = 0.0005). Time from initial symptoms to presentation to provider was longer in the younger patients (median days 23 vs. 7 for patients less than 65 vs. 65 and older, *p* = 0.0135). Other time intervals did not differ between the two age groups. Stage at diagnosis and timeliness of care did not vary significantly between patients with or without MHC in the patients less than 65 years of age or in patients 65 years of age or older (Tables7).

**Table 4:** Timeliness intervals for patients younger than 65 years with or without mental health comorbidities

	Without MHC; days (range)	With MHC; days (range)	*p*-value
Median time from symptoms to provider presentation (*N* = 105)	31 (0–3287)	13 (0–366)	0.4048
Median time from provider presentation to diagnosis (*N* = 163)	22 (0–573)	22 (0–1062)	0.7166
Median time from diagnosis to treatment (*N* = 146)	46 (0–652)	42 (0–154)	0.3635

MHC, mental health comorbidities.

**Table 5:** Timeliness intervals for patients 65 years of age and older with or without mental health comorbidities

	Without MHC; days (range) *N*	With MHC; days (range) *N*	*p*-value
Median time from symptoms to provider presentation (*N* = 141)	7 (0–1446)	4 (0–1384)	0.7197
Median time from provider presentation to diagnosis (*N* = 228)	21 (0–851)	30 (0–1062)	0.0818
Median time from diagnosis to treatment (*N* = 221)	43 (0–424)	44 (0–408)	0.7921

MHC, mental health comorbidities.

**Table 6:** Stage distribution for patients <65 with and without mental health comorbidities

Stage	Without MHC (86) *N*, %	With MHC (79)*N*, %	*p*-value
0	21 (24.42)	23 (29.11)	0.4294
I–II	21 (24.42)	13 (16.46)	
III–IV	44 (51.16)	43 (54.43)	

MHC, mental health comorbidities.

**Table 7:** Stage distribution for patients 65 and older with and without mental health comorbidities

Stage	Without MHC (160) *N*, %	With MHC (65) *N*, %	*p*-value
0	55 (34.38)	19 (29.23)	0.0567
I–II	50 (31.25)	31 (47.69)	
III–IV	55 (34.38)	15 (23.08)	

MHC, mental health comorbidities.

## Discussion

Our data show that at the VACHS, there was no difference in stage at presentation of colorectal, urothelial, and head and neck cancers between patients with or without Axis I MHC. We also found no difference in the three key measures of timeliness of care (time from onset of symptoms to presentation to a provider, time from presentation to a provider to diagnosis, and time from diagnosis to initiation of treatment) between patients with and without Axis 1 MHC. The stage distribution of colorectal and urothelial in our population was consistent with the data in the 2011 National Cancer Database 28. Our stage distribution for head and neck was comparable to other studies that looked at stage distribution for head and neck cancers in the United States and United Kingdom 29,30.

As expected 31, patients with MHC were more likely to suffer from alcohol and/or illicit drug abuse compared with the patients without MHC. Patients with MHC were also younger than patients without MHC in our cohort, likely reflecting the fact that patients with MHC tend to utilize the VA system from a younger age than patients without MHC 4,32^.^ We found no difference in stage at presentation between patients with and without MHC when we analyzed the patients below the age of 65 years and the patients 65 years of age and older separately. However, we found that younger patients presented with more advanced cancer than older patients, and the time interval between the onset of symptoms and presentation to a provider was significantly longer in younger patients. Thus, despite the fact that patients with MHC were younger than the patients without MHC, and therefore more likely to present with advanced disease and to delay care by virtue of their age, and despite the fact that they had a higher incidence of substance abuse, their stage distribution was the same as patients without MHC. This suggests that access to and utilization of care for patients with MHC at VACHS was at least as good as access to care for patients without MHC.

In contrast to our findings, most large population-based studies have shown that patients with MHC tend to present with later stage disease, and multiple studies have shown an increased cancer fatality rate for those with MHC 7,12,15,33,34. Studies of patients utilizing the VA system have shown later stage at presentation for breast and esophageal patients with MHC 16,35. Differences have been found among patients with MHC: patients with major depression were more likely to present with later stage breast cancer while those with phobia were less likely to present with later stage breast cancer 36. A study of colon cancer patients found that patients with MHC were more likely to have been diagnosed with colon cancer at autopsy, have unknown stage disease, and not to have received appropriate treatment for their cancer 37.

Timeliness of care is a one of the six measures of quality care established by the Institute of Medicine 38. However, professional organizations have not set guidelines regarding timeliness of cancer care. Most timeliness studies focus on the interval from the histologic diagnosis of malignancy to the initiation of treatment. In previous studies looking at the timeliness of lung cancer care, Schultz et al. 39 demonstrated that time to treatment for lung cancer in the VHA was comparable to wait times seen in non-VA hospitals. Merkow et al. 40, examined timeliness of care within the VHA for nonmetatstatic colon and rectal cancer and found that median time from histologic diagnosis to treatment was 27 days for colon cancer and 39 days for rectal cancer. For our combined colorectal cohort, the median time was 35.5 days. Multiple studies 41–43 have demonstrated that the quality of care within the VHA is comparable to non-VHA care both in terms of timeliness and appropriateness of cancer care. To the best of our knowledge, there have been no published studies directly looking at the impact of MHC on timeliness of cancer care. In our study, we found no evidence that patients with MHC delayed the reporting of symptoms to a provider, or were not referred for appropriate and timely workup once they reported a symptom. Thus, there was no evidence of systematic “diagnostic overshadowing,” the phenomenon by which physical complaints are attributed to underlying psychiatric disease.

It is likely that the excess mortality from cancer seen in the population-based studies discussed above is related to a lack of timely access of patients with MHC to mental and/or physical health care, resulting in less preventive care and delays in the reporting and workup of cancer-related symptoms. The lack of a negative impact of MHC on timeliness of care in our study may be a result of the unique integration of mental health services and primary care within the VHA. In order to more effectively treat patients with mental illness and significant medical comorbidities a colocated collaborative model of care was developed and implemented at the VHA starting in 2006. This model allows mental health providers to evaluate and provide treatment to patients with MHC in the setting of the primary care clinic and has been shown to increase the likelihood of diagnosing patients with MHC as well as the likelihood that these patients will follow up with subsequent mental health referrals 44,45. This care model likely improves the frequency with which patients with MHC seek out primary health care and increases the collaboration between psychiatric and primary health care providers.

In subset analyses looking at patients with alcohol or drug abuse, we did not find significant delays in care. The VA system has a higher prevalence of patients with drug and alcohol abuse and dependence than the general population 4,46 and the increased comfort and expertise of medical providers in the care of these patients, as well as the support systems for these patients in obtaining care likely contributes to their timely care.

We found that patients older than 65 had shorter intervals in presenting to a health care provider after initiation of symptoms. A similar trend has been demonstrated in lung cancer patients 47. This age-related difference could be due to increased contact with the medical community by patients over 65, increasing the likelihood that they will report worrisome symptoms. Older patients may also be more aware and more likely to discuss worrisome symptoms than younger patients.

Our study has several limitations. As it is a study of veterans, a majority of our patients (98%) were men. In addition, the median age of male veterans is older than the median age of nonveteran men 48. Because there is higher enrollment in the military during times of conflict, and therefore higher proportions of veterans from these discrete time periods, the age distribution of veterans is skewed from that of the general population 48. Our study was powered to look for stage migration, and may have been underpowered to demonstrate differences in timeliness intervals. We examined the care provided at a single VA facility, and the results may not be generalizable to other VA facilities. We determined the time interval between the date of the first symptom to the date patient first reported the symptom to a provider by retrospective chart review. However, patients do not always accurately recall and/or report the duration of their symptoms, and the provider may not always document the patient history completely or accurately. Since there is limited published data regarding timeliness of care for urothelial, colorectal, and head and neck cancer, we cannot compare our time intervals with community benchmarks. It is reassuring, however, that when care at the VA was compared to care in non-VA settings, there was increased adherence to accepted care processes and standards at the VA 49, and patients receiving care at the VA were found to have increased or equivalent survival for a variety of cancer types, including colon cancer 43. Lastly, we did not adjust for the severity of the Axis I MHC.

## Conclusions

At the VACHS, colorectal, urothelial, and head and neck cancer patients with preexisting MHC do not experience significant delays in care compared with patients without MHC. They are also not more likely to present with later stage disease than their counterparts without MHC. Our findings suggest that in an integrated system in which patients with MHC have access to comprehensive medical and psychiatric services, the presence of an Axis I MHC is not a barrier to cancer care.

## Conflict of Interest

None declared.
